# Towards precision microbiome medicine through ecological network balance

**DOI:** 10.1002/ctm2.70875

**Published:** 2026-10-27

**Authors:** Roberto Corral López, Juan A. Bonachela, Maria Gloria Dominguez‐Bello, Michael Manhart, Simon A. Levin, Martin J. Blaser, Miguel A. Muñoz

**Affiliations:** ^1^ Department of Cellular Computational and Integrative Biology University of Trento Trento Italy; ^2^ Department of Medical and Life Sciences Universitat Pompeu Fabra Barcelona Spain; ^3^ Instituto Carlos I de Física Teórica Universidad de Granada Granada Spain; ^4^ Department of Ecology, Evolution, and Natural Resources Rutgers University New Brunswick New Jersey USA; ^5^ Department of Biochemistry and Microbiology Rutgers University New Brunswick New Jersey USA; ^6^ CIFAR Humans & the Microbiome Program Toronto Canada; ^7^ Center for Advanced Biotechnology and Medicine Rutgers University Piscataway New Jersey USA; ^8^ Department of Ecology and Evolutionary Biology Princeton University Princeton New Jersey USA; ^9^ Departamento de Electromagnetismo y Física de la Materia Universidad de Granada Granada Spain

1

Dysbiosis of the gut microbiome, broadly understood as changes in community structure or function associated with impaired host health, has been linked to chronic inflammatory, metabolic, neurological, and neoplastic conditions.^1^, ^2^ However, translating these associations into robust markers that are clinically useful remains challenging. Many studies have focused on descriptive signatures, such as disease‐associated taxa, metabolic pathways, and diversity patterns, which often show inconsistent behaviour across populations and conditions.^3^, ^4^ This context‐dependence, together with a limited understanding of the ecological mechanisms underlying dysbiosis, has hindered the identification of reproducible and clinically relevant indicators for dysbiosis.^5^

To address these limitations, we recently proposed evaluating dysbiosis through community‐wide patterns of inferred microbial interactions rather than through community composition alone.^5^ We introduced a mechanistic mathematical model of gut microbial dynamics that incorporates nutrient inflow, species immigration, resource competition, and cross‐feeding (i.e. consumption of metabolites released by other species). The community emerging from these dynamics self‐organised into two distinct ecological regimes: a healthy‐like regime marked by strong temporal variability, high taxonomic and functional diversity, and predominantly negative interactions; and a dysbiotic‐like regime characterised by more persistent, metabolically inter‐connected consortia, and a shift toward more positive interactions.^5^

This observation motivated the creation of an ecological network balance index (ENBI), which quantifies the balance of positive and negative inferred effective interactions relative to healthy states of the community. A positive ENBI indicates a shift toward effective positive interactions (regardless of whether they dominate). In simulated communities, the ENBI tracked the transition between healthy‐like and dysbiotic‐like regimes. Importantly, in clinical cohorts with available metagenomic data, the ENBI showed consistent disease‐associated increases across inflammatory bowel disease, irritable bowel syndrome, *Clostridioides difficile* infection, and colorectal cancer (CRC). Furthermore, in CRC, ENBI values increased with clinical stage, suggesting that interaction balance has the potential to reflect disease progression.^5^ We are currently extending these analyses to additional diseases and datasets, with preliminary findings confirming and broadening the relevance of this pattern.

The translational value of this community metric is threefold (Figure 1): its consistency across diseases, its potential for longitudinal monitoring, and the new perspective it offers for interpreting metagenomic data and designing targeted interventions. The consistency of the ENBI may reflect its ability to capture a more fundamental ecological feature of dysbiosis: although disease‐associated taxa or functions can vary across conditions and even between cohorts of people with the same disease,^3^, ^4^ the conserved qualitative changes of the respective communities (i.e. the shift in interaction balance) suggest a conserved dysbiosis‐related ecological reorganisation. This conservation enables the use of the ENBI as a way to assess disease risk in a noninvasive way, by using stool metagenomic sequencing. In particular, the association observed between the ENBI and CRC stage motivates prospective studies testing whether the metric tracks disease progression in other conditions. Such studies could establish whether longitudinal changes in the patient's ENBI help detect emerging dysbiosis, follow disease trajectories and responses to therapies, or monitor ecological recovery after antibiotic treatment, inflammatory flares, or microbiome‐targeted interventions.

**FIGURE 1:**
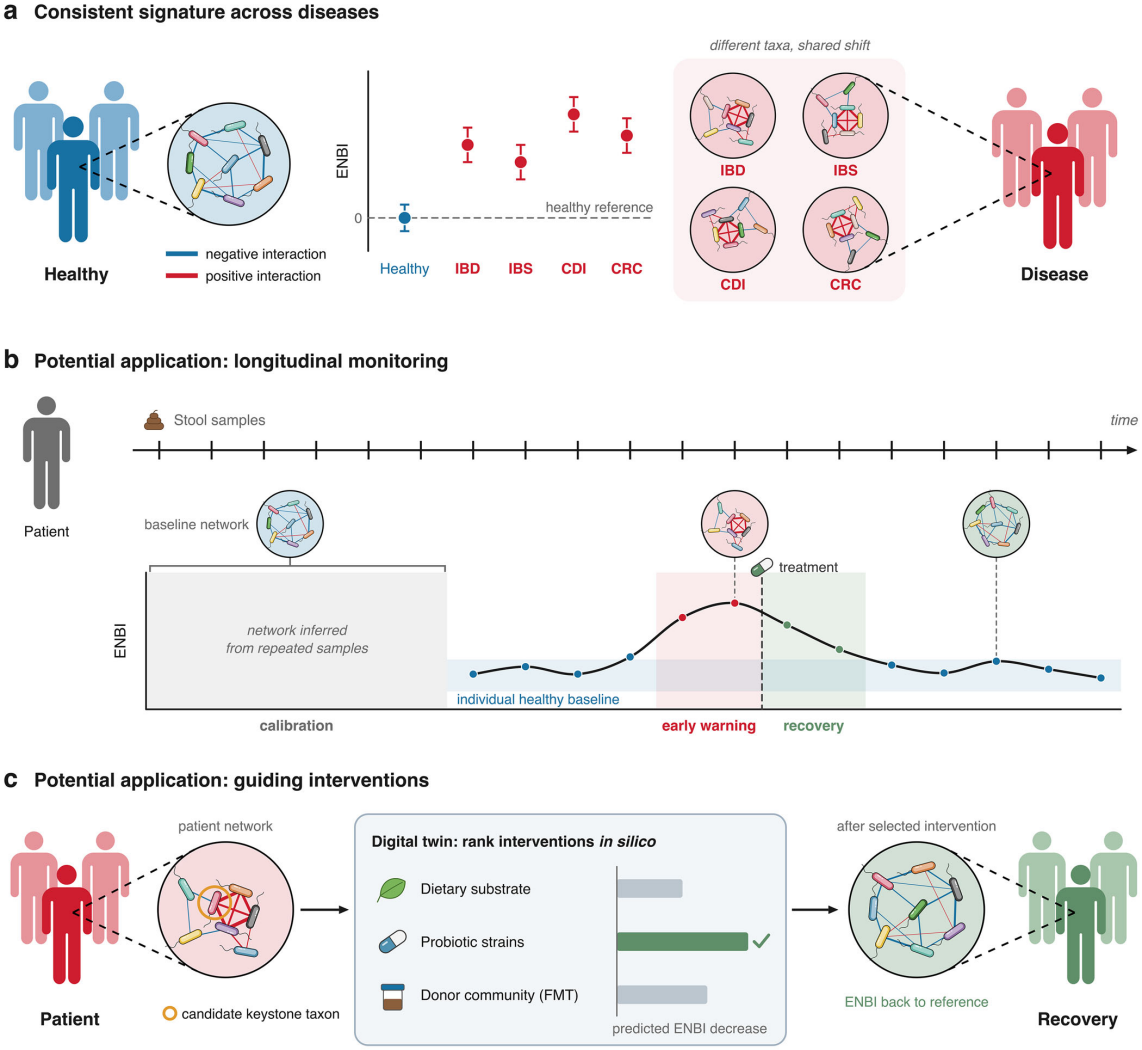
WE HAVE AN IMPROVED VERSION OF THE FIGURE. WE ATTACH IT IN PNG FORMAT. Translational uses of the ecological network balance index (ENBI). (a) In inflammatory bowel disease (IBD), irritable bowel syndrome (IBS), *Clostridioides difficile* infection (CDI) and colorectal cancer (CRC), gut communities differ in composition but share a shift toward positive inferred interactions, which raises the ENBI relative to healthy reference networks.^5^ (b) Potential future application to longitudinal monitoring: repeated stool samples collected during an initial calibration period could support inference of an individual's baseline interaction network. Updating this network with subsequent samples would allow ENBI to flag departures from that baseline and track recovery after treatment. (c) Potential future application to intervention design: effective interaction networks could help identify candidate keystone taxa and interactions that may sustain dysbiosis. A digital twin calibrated to the patient would rank candidate interventions (substrates, strains, or donor communities) in silico, guiding subsequent experimental and clinical testing. Blue and red links represent, respectively, negative and positive inferred interactions; link width represents interaction strength. Values are only for illustration purposes.

Analysis of effective interaction networks underlying the gut microbiome could help clarify the ecological roles of individual species in sustaining dysbiotic communities and identify interactions that might be targeted (i.e. fostered or hindered) to support recovery. Such analyses may reveal candidate keystone taxa whose ecological importance is not apparent from differential abundance alone.^6^ A mechanistic precedent comes from work combining ecological modelling and experiments, which showed that *Clostridium scindens* enhances resistance to *C. difficile* in mice.^7^

Despite advances in microbiome‐targeted interventions—including diet, prebiotics, probiotics and faecal microbiota transplantation (FMT)—predicting responses at the level of the individual patient remains challenging.^8^, ^9^ Moreover, the ecological indicators of treatment success are not fully understood or lack consistency. For instance, donor‐strain engraftment varies across recipients and diseases,^10^ and a single‐arm trial combining FMT with anti–PD‐1‐based immunotherapy reported clinical benefit in only a subset of patients with refractory melanoma.^11^ Our ecological metric could be used to test whether responders and non‐responders differ in their initial network organisation, and whether treatment‐associated changes in interaction balance accompany clinical improvement. We are currently testing the ENBI's behaviour across intervention datasets, with encouraging preliminary results in immunotherapy cohorts.

The information above can be used to link the ecological roles of key taxa to the effects of specific interventions, which could provide a basis for precision microbiome medicine; for example, by enabling a patient‐specific selection of interventions that specifically modify the interactions that sustain dysbiosis. The latter can be safely achieved by building a digital twin of the patient's gut microbial ecosystem, calibrated to their metagenomic and metabolomic profiles.^12^ Such a model could be used to rank candidate substrates, strains, or donor communities in silico according to their ability to modify (in silico) interactions and support recovery, information that then can be used to guide subsequent experimental and clinical testing.

Translation, nevertheless, requires resolving several limitations associated with our metric. For example, inferred interactions represent effective relationships that may reflect indirect microbial effects, shared environmental responses, or host influences, and are sensitive to compositional artefacts, taxonomic profiling, and inference choices.^13^, ^14^ The sign of an inferred interaction therefore does not uniquely identify specific metabolic exchanges; moreover, because different inferred networks can produce similar ENBI values, the index alone cannot identify which strain, substrate, or donor community should be selected. Complementary approaches could help connect these inferred patterns to underlying mechanisms: genome‐scale metabolic models can predict feasible exchanges,^12^ while experiments in gut simulators such as SHIME could assess how controlled changes in nutrient supply and other environmental factors affect community organisation and the ENBI.^15^ In addition, the ENBI is currently a cohort‐level quantity, derived from networks that are inferred across multiple samples and is not yet validated for assessment of individual patients. Potential routes toward individual estimates include longitudinal sampling of the same individual and comparison with large, well‐characterised reference datasets stratified by clinical condition. As a final caveat, the ENBI does not establish disease aetiology; its clinical role would be to complement disease‐specific assessments and established diagnostic protocols.

Analysing the underlying effective interaction networks offers a starting point for connecting a shared ecological signature of dysbiosis, the ENBI, with potential targets for intervention. Establishing the clinical value of this approach will require prospective studies combining standardised network inference with metabolomic, host measurements and clinical follow‐up, while accounting for diet, medication and other confounders. Demonstrating that this ecological information improves clinical decisions and patient outcomes would help advance precision microbiome medicine, with the specified interventions tailored to the organisation and functional dependencies of each patient's microbial community.

## FUNDING INFORMATION

This work has been supported by Grant No. PID2023‐149174NB‐I00 financed by the Spanish Ministry and Agencia Estatal de Investigación MICIU/AEI/10.13039/501100011033 and EDRF funds. It has also supported by a Simons Early Career Investigator in Marine Microbial Ecology and Evolution Award (award no.826106), National Science Foundation grant DMS‐2052616 to J.A.B. Finally, R.C.L. is supported by an EMBO Postdoctoral Fellowship (ALTF 190‐2026).

## References

[1] Hou K , Wu Z‐X , Chen X‐Yu , et al. Microbiota in health and diseases. Signal Transduct Target Ther. 2022;7:135. doi: 10.1038/s41392-022-00974-4 PMC9034083 35461318

[2] Li Y , Xiao X , Jiang X , Li X , Wang W , Lin R . Gut microbiota in health and disease. Mol Biomed. 2026;7:116. doi: 10.1186/s43556-026-00512-6 PMC13385334 42474869

[3] Duvallet C , Gibbons SM , Gurry T , Irizarry RA , Alm EJ . Meta‐analysis of gut microbiome studies identifies disease‐specific and shared responses. Nat Commun. 2017;8:1784. doi: 10.1038/s41467-017-01973-8 PMC5716994 29209090

[4] He Y , Wu W , Zheng H‐M , et al. Regional variation limits applications of healthy gut microbiome reference ranges and disease models. Nat Med. 2018;24:1532‐1535. doi: 10.1038/s41591-018-0164-x 30150716

[5] Corral López R , Bonachela JA , Dominguez‐Bello MG , et al. Imbalance in gut microbial interactions as a marker of health and disease. Science. 2026;391:890‐895. doi: 10.1126/science.ady1729 41747050

[6] Banerjee S , Schlaeppi K , van der Heijden MGA . Keystone taxa as drivers of microbiome structure and functioning. Nat Rev Microbiol. 2018;16:567‐576. doi: 10.1038/s41579-018-0024-1 29789680

[7] Buffie CG , Bucci V , Stein RR , et al. Precision microbiome reconstitution restores bile acid mediated resistance to *Clostridium difficile* . Nature. 2015;517:205‐208. doi: 10.1038/nature13828 PMC4354891 25337874

[8] Zmora N , Zilberman‐Schapira G , Suez J , et al. Personalized gut mucosal colonization resistance to empiric probiotics is associated with unique host and microbiome features. Cell. 2018;174:1388‐1405.e21. doi: 10.1016/j.cell.2018.08.041 e21 30193112

[9] Lee KA , Thomas AM , Bolte LA , et al. Cross‐cohort gut microbiome associations with immune checkpoint inhibitor response in advanced melanoma. Nat Med. 2022;28:535‐544. doi: 10.1038/s41591-022-01695-5 PMC8938272 35228751

[10] Ianiro G , Punčochář M , Karcher N , et al. Variability of strain engraftment and predictability of microbiome composition after fecal microbiota transplantation across different diseases. Nat Med. 2022;28:1913‐1923. doi: 10.1038/s41591-022-01964-3 PMC9499858 36109637

[11] Davar D , Dzutsev AK , McCulloch JA , et al. Fecal microbiota transplant overcomes resistance to anti–PD‐1 therapy in melanoma patients. Science. 2021;371:595‐602. doi: 10.1126/science.abf3363 PMC8097968 33542131

[12] Mardinoglu A , Palsson BØ . Genome‐scale models in human metabologenomics. Nat Rev Genet. 2025;26:123‐140. doi: 10.1038/s41576-024-00768-0 39300314

[13] Weiss S , Van Treuren W , Lozupone C , et al. Correlation detection strategies in microbial data sets vary widely in sensitivity and precision. ISME J. 2016;10:1669‐1681. doi: 10.1038/ismej.2015.235 PMC4918442 26905627

[14] Carr A , Diener C , Baliga NS , Gibbons SM . Use and abuse of correlation analyses in microbial ecology. ISME J. 2019;13:2647‐2655. doi: 10.1038/s41396-019-0459-z PMC6794304 31253856

[15] Molly K , Vande Woestyne M , Verstraete W . Development of a 5‐step multi‐chamber reactor as a simulation of the human intestinal microbial ecosystem. Appl Microbiol Biotechnol. 1993;39:254‐258. doi: 10.1007/BF00228615 7763732

